# Rice ubiquitin‐conjugating enzyme OsUBC26 is essential for immunity to the blast fungus *Magnaporthe oryzae*

**DOI:** 10.1111/mpp.13132

**Published:** 2021-08-30

**Authors:** Xin Liu, Linlin Song, Heng Zhang, Yijuan Lin, Xiaolei Shen, Jiayuan Guo, Meiling Su, Gaosheng Shi, Zonghua Wang, Guo‐Dong Lu

**Affiliations:** ^1^ State Key Laboratory of Ecological Pest Control for Fujian and Taiwan Crops Key Laboratory of Biopesticide and Chemistry Biology Ministry of Education Fujian Agriculture and Forestry University Fuzhou China

**Keywords:** AvrPiz‐t, *Magnaporthe oryzae*, OsUBC26, plant immunity, rice, ubiquitin‐conjugating enzyme, WRKY45

## Abstract

The functions of ubiquitin‐conjugating enzymes (E2) in plant immunity are not well understood. In this study, OsUBC26, a rice ubiquitin‐conjugating enzyme, was characterized in the defence against *Magnaporthe oryzae*. The expression of *OsUBC26* was induced by *M. oryzae* inoculation and methyl jasmonate treatment. Both RNA interference lines and CRISPR/Cas9 null mutants of *OsUBC26* reduced rice resistance to *M. oryzae*. *WRKY45* was down‐regulated in *OsUBC26* null mutants. In vitro E2 activity assay indicated that OsUBC26 is an active ubiquitin‐conjugating enzyme. Yeast two‐hybrid assays using OsUBC26 as bait identified the RING‐type E3 ligase UCIP2 as an interacting protein. Coimmunoprecipitation assays confirmed the interaction between OsUBC26 and UCIP2. The CRISPR/Cas9 mutants of *UCIP2* also showed compromised resistance to *M. oryzae*. Yeast two‐hybrid screening using UCIP2 as bait revealed that APIP6 is a binding partner of UCIP2. Moreover, OsUBC26 working with APIP6 ubiquitinateds AvrPiz‐t, an avirulence effector of *M. oryzae*, and *OsUBC26* null mutation impaired the proteasome degradation of AvrPiz‐t in rice cells. In summary, OsUBC26 plays important roles in rice disease resistance by regulating *WRKY45* expression and working with E3 ligases such as APIP6 to counteract the effector protein AvrPiz‐t from *M. oryzae*.

## Graphical abstract

Rice ubiquitin‐conjugating enzyme OsUBC26 is essential for immunity to blast fungus by regulating *WRKY45* expression and working with APIP6 to promote the ubiquitination and degradation of AvrPiz‐t.

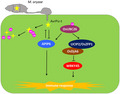

## 1 INTRODUCTION

The ubiquitin‐26S proteasome system (UPS) involves the sequential activities of three enzymes, namely ubiquitin activating enzyme (E1), ubiquitin‐conjugating enzyme (E2), and ubiquitin protein ligase enzyme (E3). First, ubiquitin is activated by E1 in an ATP‐dependent manner. Then, the ubiquitin (Ub)‐tagged E1 delivers Ub to E2 through transesterification. Ultimately, the ubiquitinated E2 targets E3, which recruits substrates for ubiquitination (Hershko, 2005; Smalle & Vierstra, 2004).

In plants, ubiquitination plays critical roles in responses to biotic and abiotic stimuli (Dielen et al., 2010; Marino et al., 2012; Stone, 2014; Zeng et al., 2006). During rice–pathogen interactions, E3 ubiquitin ligase plays an important role to counteract pathogens (Ning et al., 2016). For example, rice Spl11 is an E3 ligase and functions as a negative regulator of programmed cell death and disease resistance (Zeng et al., 2004). Rice APIP6 and APIP10 are RING‐type E3 ligases that interact with AvrPiz‐t effector from *Magnaporthe oryzae* and promote the ubiquitination and degradation of AvrPiz‐t, therefore positively regulating rice immunity to blast fungus (Park et al., 2012, 2016). Rice Xa21‐binding protein 3 (XB3) is another RING‐type E3 ligase that is required in Xa21‐mediated resistance to *Xanthomonas oryzae* pv. *oryzae* (Wang et al., 2006).

In addition to ubiquitin E3 ligases, a number of plant E2 genes have been identified and functionally characterized in diverse biological processes. The overexpression of *VrUBC1*, *AhUBC2*, and *GmUBC2* enhanced the drought tolerance of transgenic *Arabidopsis thaliana* (Chung et al., 2013; Wan et al., 2011; Zhou et al., 2010). Ectopic expression of wild rice, *Oryza grandiglumis,* ubiquitin‐conjugating enzyme 1 (*OgUBC1)* in *Arabidopsis* increases resistance to *Botrytis cinerea* infection (Jeon et al., 2012). *Triticum aestivum* ubiquitin‐conjugating enzyme 4 (TaU4) is a negative regulator of wheat defence against *Septoria*, and virus‐induced gene silencing of *TaU4* results in delayed disease symptom development (Millyard et al., 2016). A group of E2 enzymes in tomato is essential for plant immunity, and gene silencing of the homologs in tobacco reduced pattern‐triggered immunity (Zhou et al., 2017). Although these reports indicate that E2 enzymes are involved in plant defence, their specific mechanisms still need to be revealed.

There are 48 members of E2s in rice plants that have been classified into 15 groups according to *Arabidopsis* and human E2s (Bae & Kim, 2014). However, none of them have been characterized in rice immunity. In this study, we characterize a group VII E2 enzyme, OsUBC26, in rice immunity by using reverse genetic and biochemical methods. CRISPR/Cas9 mutants of *OsUBC26* significantly compromised rice resistance to *M. oryzae*. We also screened a rice cDNA library and found that RING‐type E3 ligase UCIP2 is a working partner of OsUBC26. In addition, OsUBC26 could work with APIP6 and ubiquitinate AvrPiz‐t in vitro. Our work revealed a ubiquitin‐conjugating enzyme in rice that is essential for rice immunity.

## 2 RESULTS

### 2.1 *OsUBC26* expression was induced by *M. oryzae* infection and methyl jasmonate treatment

**FIGURE 1:**
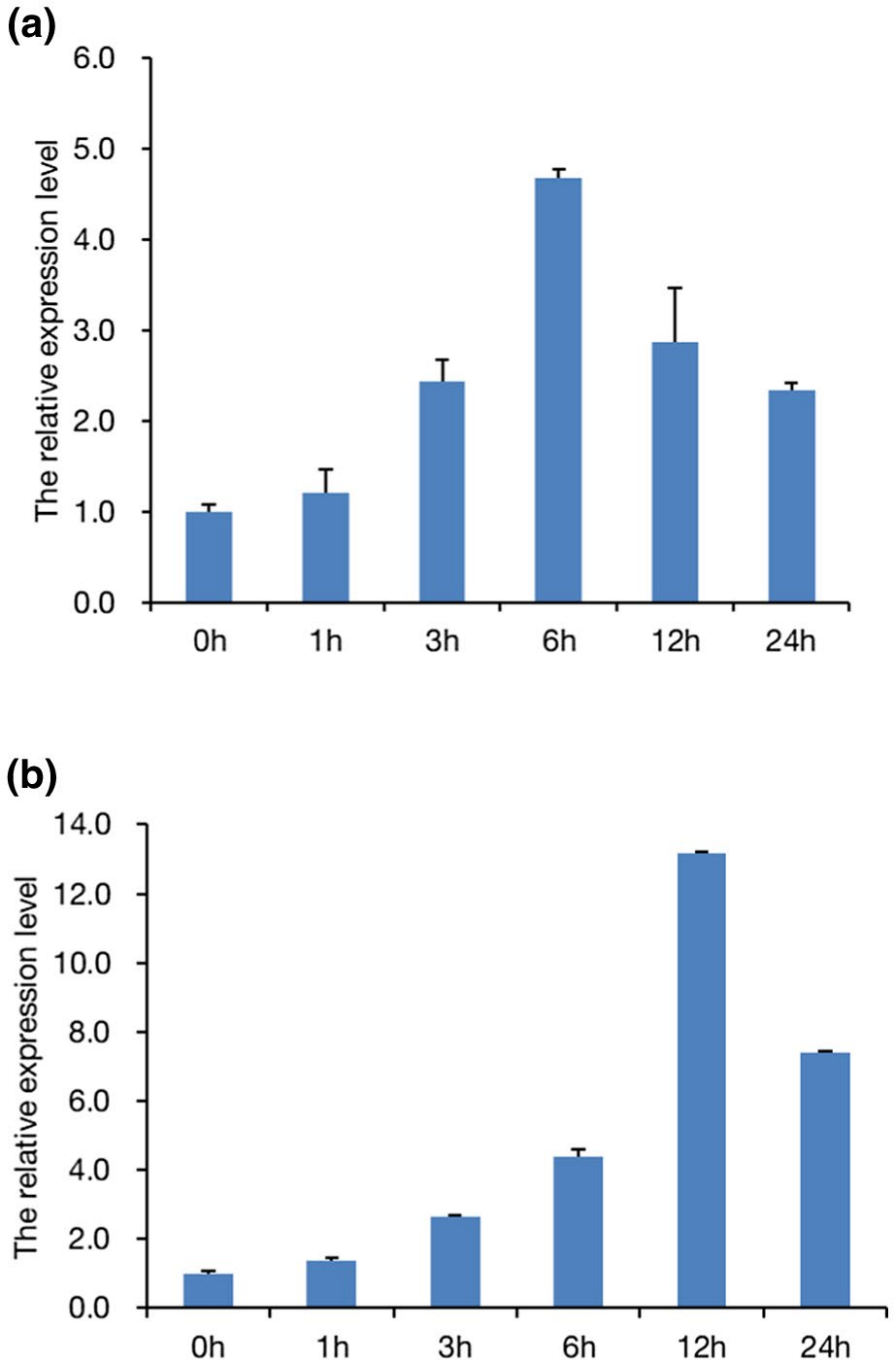
The expression of *OsUBC26* was induced by *Magnaporthe oryzae* inoculation and methyl jasmonate (Me‐JA) treatment. (a) Expression pattern of *OsUBC26* after inoculation with Guy11. (b) Expression pattern of *OsUBC26* after treatment with 100 μM Me‐JA. Time is hours post‐treatment (hpt). Each experiment was done at least three times. Values are the means of three replicates and the error bars represent *SEM*

Our previous study indicated that the expression of *OsUBC26* can be induced by *M. oryzae*, *X*. *oryzae* pv. *oryzae*, and *Rhizoctonia solani* infection (Zhao et al., 2008). To further investigate the expression profile of *OsUBC26*, quantitative reverse transcription PCR (RT‐qPCR) was used to quantify the expression level of *OsUBC26* at different time points during rice blast infection. As illustrated in Figure 1a, *OsUBC26* responds to rice blast fungus from a very early stage, as the expression level of *OsUBC26* increased quickly after the challenge by *M. oryzae* with a peak at 6 hr postinoculation (hpi) and then gradually decreased. As salicylic acid (SA) and jasmonic acid (JA) are two major phytohormones involved in disease resistance, SA and methyl jasmonate (Me‐JA) were used to treat rice seedlings. We found that SA had no obvious effect on the expression of *OsUBC26* (data not shown). On the other hand, the expression of *OsUBC26* increased to the highest level at 12 hours post‐treatment (hpt) and was approximately 13 times higher than the expression level observed at 0 hpt after the Me‐JA treatment (Figure 1b). This result suggests that *OsUBC26* selectively participates in the Me‐JA‐mediated defence response of rice.

### 2.2 OsUBC26 is important for rice immunity against *M. oryzae* infection

**FIGURE 2:**
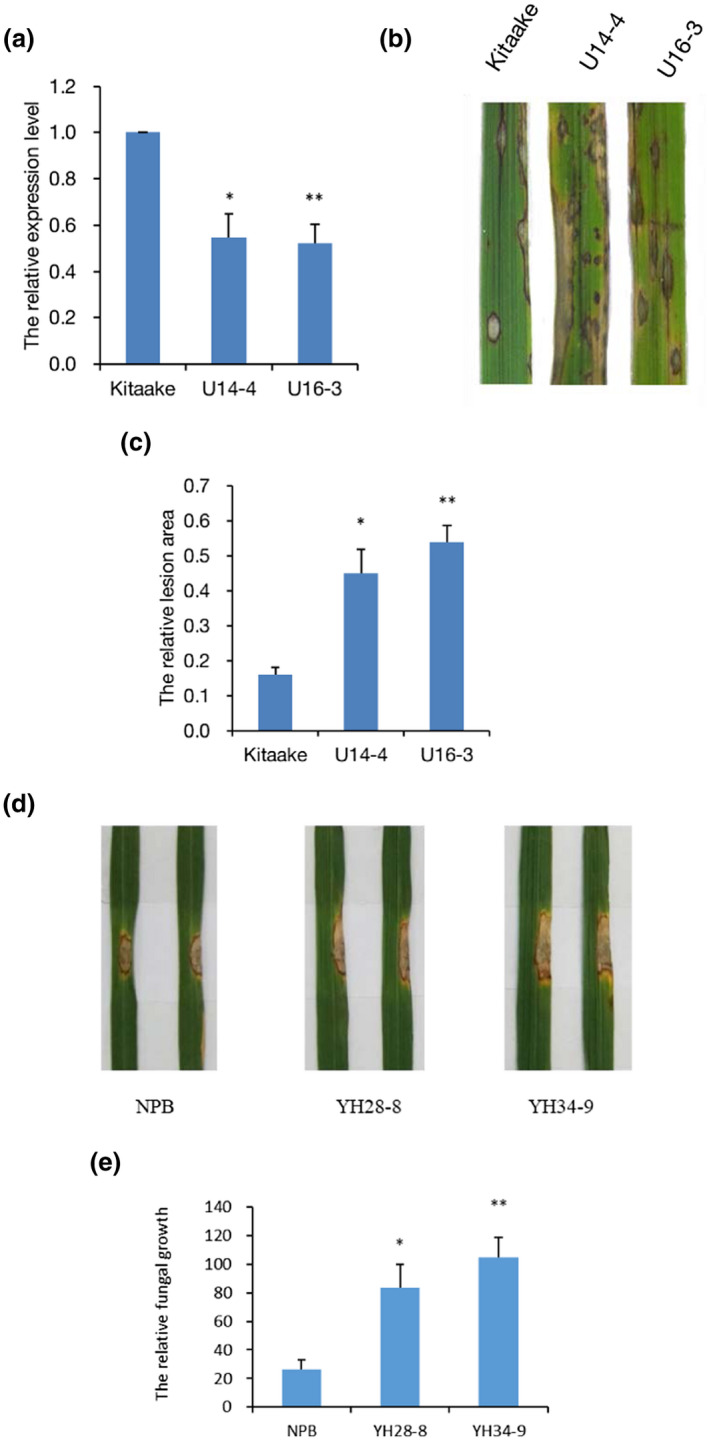
*OsUBC26* RNAi and null mutation reduce rice resistance to *Magnaporthe oryzae*. (a) The relative expression level of *OsUBC26* in its RNAi lines. (b) Inoculation results of *OsUBC26* RNAi lines and corresponding wild‐type plants. Three‐week‐old rice seedlings were inoculated with a conidial spore suspension of *M. oryzae* isolate Guy11. Pictures were taken 7 days postinoculation (dpi). (c) Relative lesion area of RNAi lines and corresponding wild‐type plants. The relative lesion area was measured by ImageJ software. (d) Punch inoculation of *OsUBC26* null mutants and its background cultivar Nipponbare (NPB). Leaves of 6‐week‐old rice plants were inoculated with virulent isolate FJ86‐CT. The leaves were photographed at 7 dpi. (e) Relative fungal growth (2^[^ *^C^* ^t(^ *^OsUBQ^* ^)−^ *^C^* ^t(^ *^MoPOT^* ^2)]^ × 100) was measured at 7 dpi. Each experiment was done at least three times. Values are the means of three replicates and error bars represent *SEM*. Significant difference, *p < 0.05, **p < 0.01

To characterize the role of OsUBC26 in rice resistance to *M. oryzae OsUBC26* RNAi lines were generated in the background of Kitaake. Transcription analysis showed that the expression of *OsUBC26* in the RNAi lines was approximately half that of wild‐type plants (Figure 2a). *M. oryzae* inoculation assay demonstrated that the disease symptoms became more severe in *OsUBC26* RNAi lines with a larger lesion area than wild‐type plants (Figure 2b,c). Hence, RNA interference of *OsUBC26* diminished rice resistance to *M. oryzae*. To further characterize the function of OsUBC26, gene null mutants were generated by CRISPR/Cas9 technology. A 2 bp deletion and a 1 bp deletion in target 1 cause a premature stop codon (Figure S1). Homozygous mutants were selected in the T_3_ generation. The punch inoculation method was used for disease resistance evaluation (Ono et al., 2001; Park et al., 2012). The result showed that the lesion size and relative fungal biomass were significantly larger and greater in *OsUBC26* null mutants compared to wild‐type Nipponbare (Figure 2d,e). Thus, *OsUBC26* is an important factor for rice immunity against *M. oryzae* infection.

### 2.3 Null mutation of *OsUBC26* down‐regulated the expression of *WRKY45*

**FIGURE 3:**
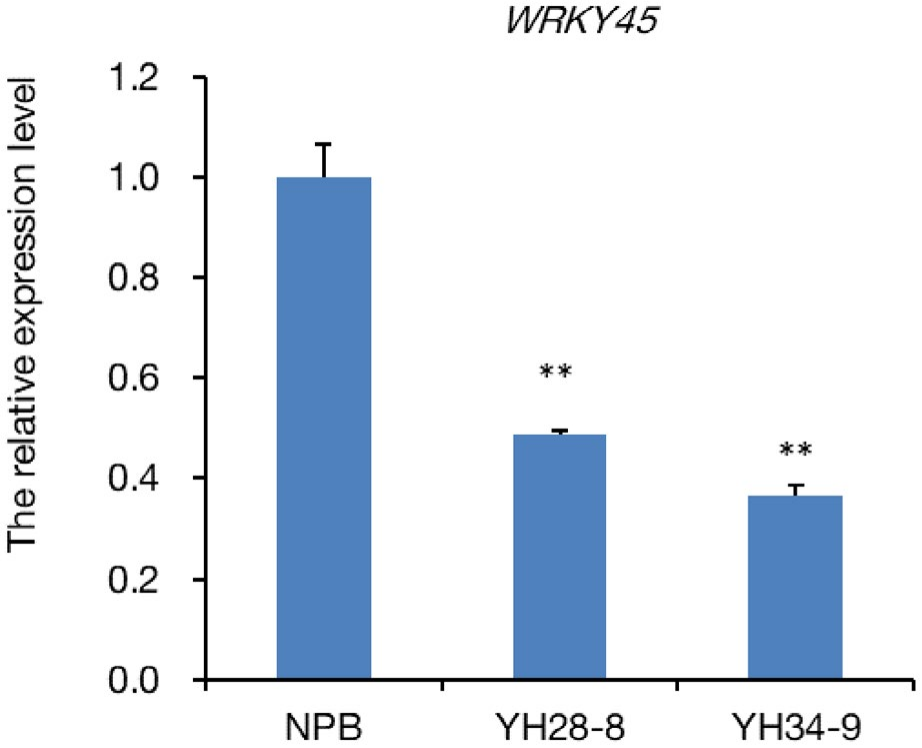
*WRKY45* is down‐regulated in *OsUBC26* null mutants. Values are the means of three replicates and error bars represent *SEM*. Significant difference, **p < 0.01

To reveal why CRISPR/Cas9 null mutants of *OsUBC26* compromised rice resistance to *M. oryzae*, RNA‐Seq of the *OsUBC26* null mutant and its wild‐type Nipponbare was conducted. A total of 237 genes were up‐regulated and 215 genes were down‐regulated in the *OsUBC26* null mutant (Tables S1 and S2). The lower expression of *WRKY45* in OsUBC26 null mutant attracted our attention. *WRKY45* is a WRKY transcription factor that positively regulates rice resistance to the blast fungus and its function is affected by nuclear ubiquitin proteasome degradation (Matsushita et al., 2013; Shimono et al., 2007, 2012). The down‐regulation of *WRKY45* in the *OsUBC26* null mutants was further confirmed by RT‐qPCR (Figure 3).

### 2.4 OsUBC26 is an active ubiquitin‐conjugating enzyme

**FIGURE 4:**
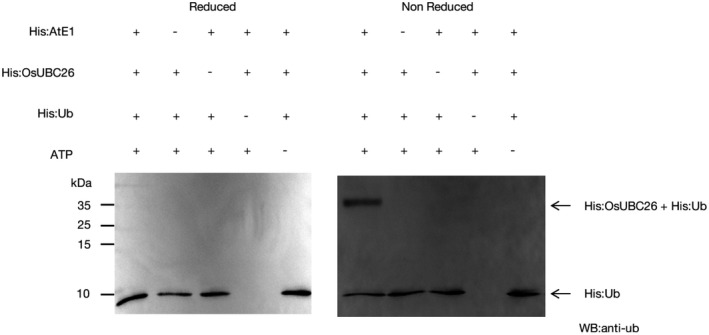
In vitro E2 activity assay of OsUBC26. E2 charging assays of His:OsUBC26 under nonreduced and reduced conditions. Ubiquitin bound to OsUBC26 under nonreduced conditions but not reduced conditions

In the presence of ATP, ubiquitin is activated by ubiquitin activating enzyme (E1), then transferred to the active site cysteine in E2 to form a thioester bond between ubiquitin and E2 (Smalle & Vierstra, 2004; Varshavsky, 1997; Wenzel et al., 2011; Ye & Rape, 2009). This thioester bond is sensitive to the thiol‐reducing agent dithiothreitol (DTT) and this characteristic can be used for E2 activity assay (Kraft et al., 2005). In an effort to prove that OsUBC26 is an active ubiquitin‐conjugating enzyme, histidine‐tagged *Arabidopsis* E1 component His:AtE1 and ubiquitin component His:ubiquitin, a histidine‐tagged rice E2 component His:OsUBC26, were expressed in a prokaryotic expression system and purified separately. Under nonreduced (without DTT) and reduced (with DTT) conditions, western blot analysis demonstrated that ubiquitin bound to His:OsUBC26 under nonreduced conditions, but not reduced conditions (Figure 4). This experiment demonstrates that OsUBC26 is an active ubiquitin‐conjugating enzyme.

### 2.5 OsUBC26 interacts with UCIP2

**FIGURE 5:**
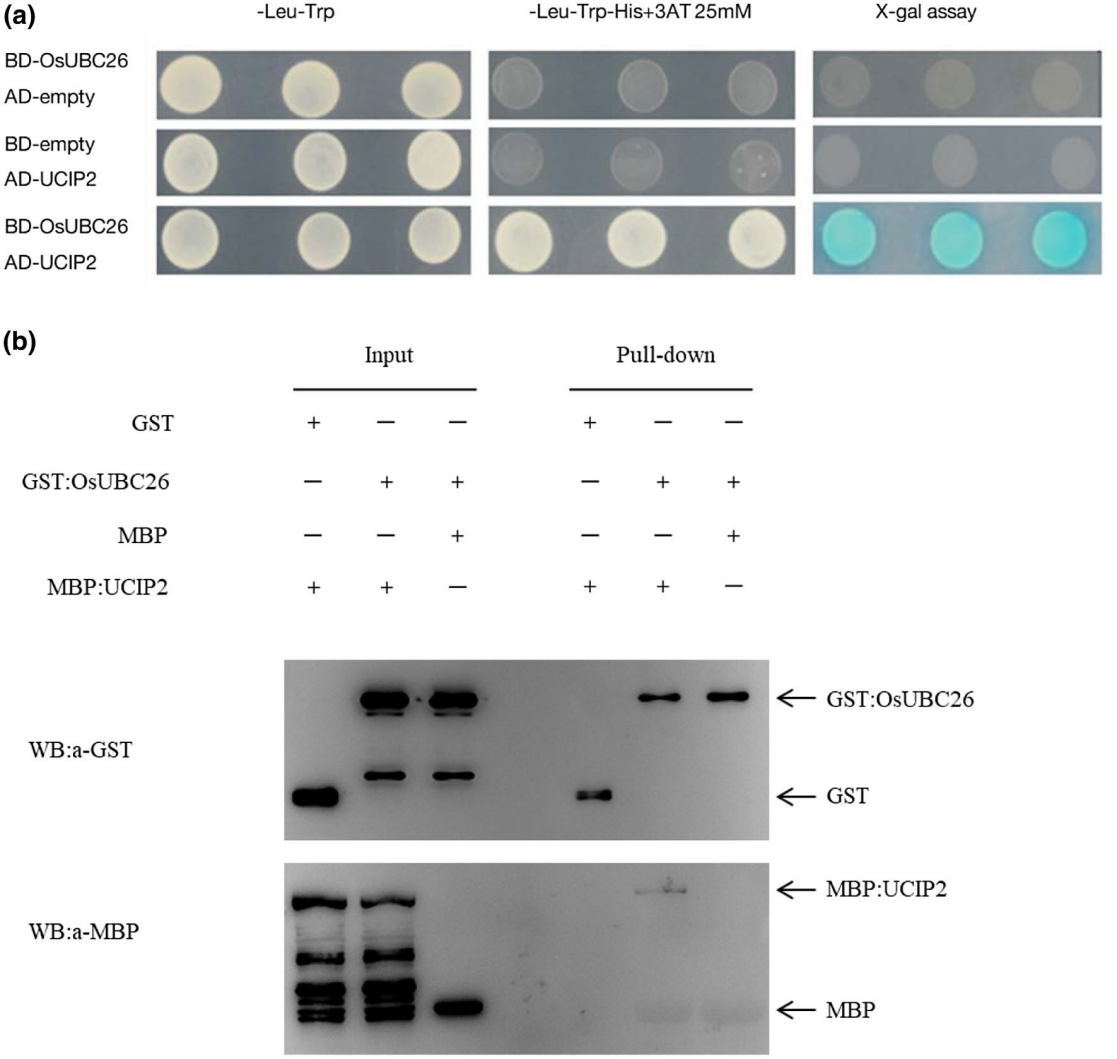
OsUBC26 interacts with UCIP2. (a) OsUBC26 interacts with UCIP2 in yeast two‐hybrid assay. The full‐length coding sequence of *UCIP2* was cloned and fused to the GAL4‐activation domain. Cotransformation with BD‐UBC26 further confirmed the interaction between OsUBC26 and UCIP2. (b) OsUBC26 interacts with UCIP2 in glutathione S‐transferase (GST) pull‐down assay. GST‐tagged OsUBC26 was expressed in *Escherichia coli* and conjugated to glutathione magarose beads, which were used for maltose‐binding protein (MBP):UCIP2 pull‐down assay

To further reveal the working partner of OsUBC26, a yeast two‐hybrid screening was conducted using OsUBC26 as bait. Several E3 ligases that interact with OsUBC26 were identified from a rice cDNA library in the yeast two‐hybrid assay (Table 1). Among them, RING‐type E3 ligase UCIP2 (OsUBC26 Interact Protein 2) represented 17 of 44 of the identified interacting clones. We cloned the full‐length coding sequence of UCIP2 and confirmed the interaction between OsUBC26 and UCIP2 in yeast two‐hybrid assay (Figure 5a). In addition, glutathione S‐transferase (GST)‐tagged OsUBC26 and maltose‐binding protein (MBP)‐tagged UCIP2 were prepared in a prokaryotic expression system. A GST pull‐down assay showed that GST:OsUBC26 interacted with MBP:UCIP2 (Figure 5b). Thus, OsUBC26 may have preference for UCIP2 and mediated the ubiquitination degradation of UCIP2‐recruited substrates. Interestingly, the protein ID of UCIP2 (LOC_Os01g47740) is the same as OsZFP1, which has been reported as a working partner of OsDjA6 (Zhong et al., 2018). OsDjA6, a chaperone DnaJ protein, negatively regulates *WRKY45* expression and rice immunity to *M. oryzae* (Zhong et al., 2018). Thus, OsUBC26 as a conjugating enzyme works with E3 ligase UCIP2/OsZFP1 and chaperone DnaJ protein OsDjA6 to modulate rice innate immunity through regulation of *WRKY45* expression.

**TABLE 1:** Summary of OsUBC26‐interacting proteins identified by yeast two‐hybrid screening

Gene Code	Gene_locus	Gene_description	No. of independent clones
UCIP1	LOC_Os08g42640	Zinc finger, RING‐type domain containing protein	3
UCIP2	LOC_Os01g47740	Zinc finger, RING‐type domain containing protein	17
UCIP3	LOC_Os04g10680	Similar to protein containing C‐terminal RING‐finger	2
UCIP4	LOC_Os09g33670	Zinc finger, RING‐type domain containing protein	5
UCIP5	LOC_Os12g39640	Similar to transfactor‐like protein (MYB)	1
UCIP6	LOC_Os02g44599	Protein kinase‐like domain containing protein	1
UCIP7	LOC_Os04g47770	Similar to *cis*‐zeatin *O*‐glucosyltransferase 1	1
UCIP8	LOC_Os03g44900	Ccr4‐Not complex, subunits 3 and 5 family protein	1
UCIP9	LOC_Os04g33590	Esterase/lipase/thioesterase domain containing protein	2
UCIP10	LOC_Os01g56580	Similar to casein kinase‐like protein	1
UCIP11	LOC_Os03g63950	Similar to plastid‐specific 30S ribosomal protein1	1
UCIP12	LOC_Os12g31850	Similar to ureide permease 1 (AtUPS1)	1
UCIP13	LOC_Os10g27340	Prolyl 4‐hydroxylase, alpha subunit domain containing protein	1
UCIP14	LOC_Os06g07350	Similar to RNA‐binding protein‐like	1
UCIP15	LOC_Os05g48360	Membrane attack complex component/perforin/ complement C9 family protein	1
UCIP16	LOC_Os08g26870	Protein of unknown function DUF151 domain containing protein	1
UCIP17	LOC_Os08g44820	Similar to CUC2	1
UCIP18	LOC_Os01g52110	RING‐type domain containing protein	1
UCIP19	LOC_Os06g06760	U‐box domain containing protein	2

### 2.6 *UCIP2* plays a positive role in resistance to *M. oryzae*

**FIGURE 6:**
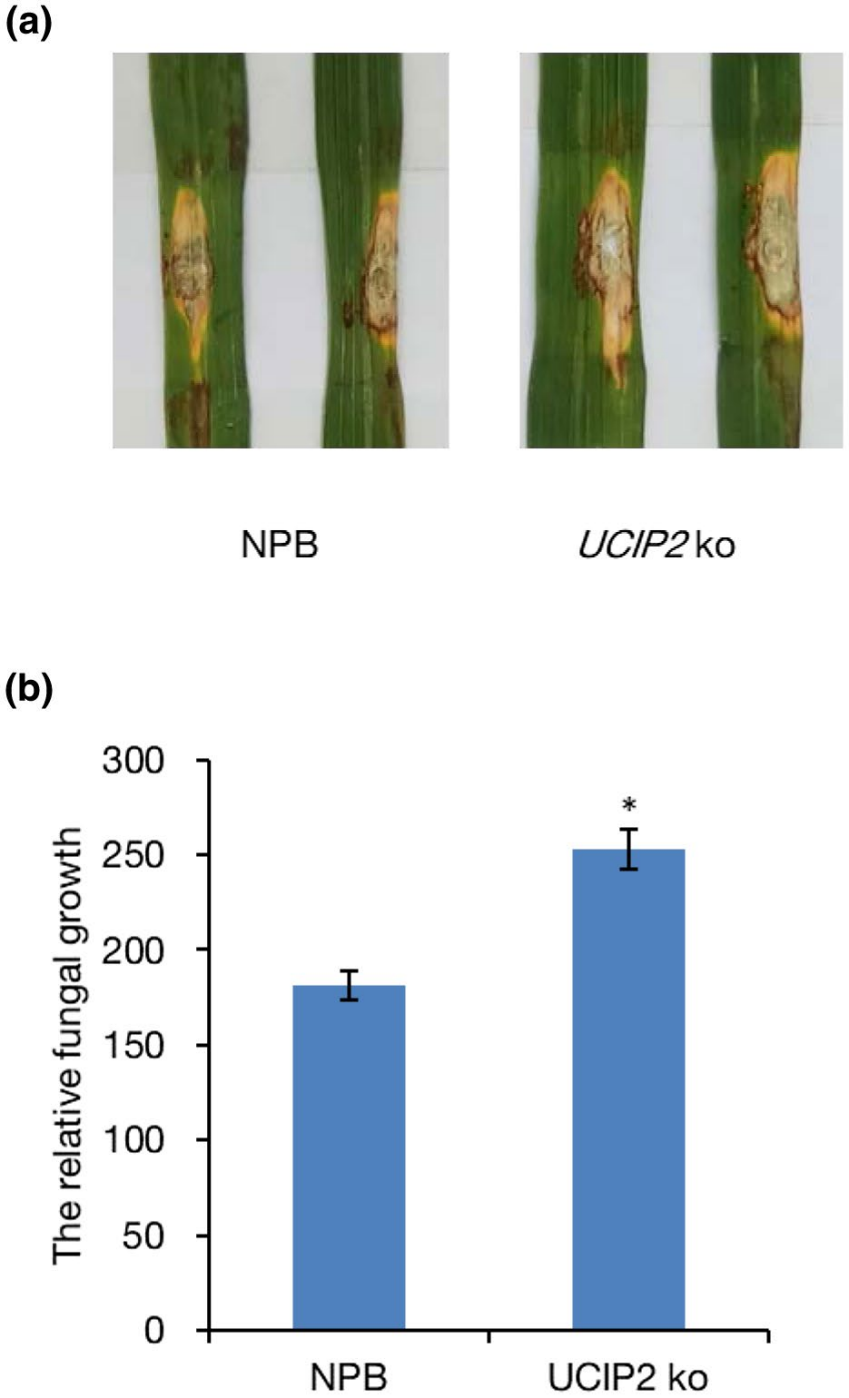
Null mutation of *UCIP2* compromises resistance to *Magnaporthe oryzae*. (a) Punch inoculation of *UCIP2* null mutants and its background cultivar Nipponbare (NPB). Leaves of 6‐week‐old rice plants were inoculated with virulent isolate FJ86‐CT. The leaves were photographed at 7 days postinoculation (dpi). (b) Relative fungal growth (2^[^ *^C^* ^t(^ *^OsUBQ^* ^)−^ *^C^* ^t(^ *^MoPOT^* ^2)]^ × 100] was measured at 7 dpi. Values are the means of three replicates and error bars represent *SEM*. Significant difference, *p < 0.05

To reveal the role of UCIP2 in rice immunity to *M. oryzae*, the CRISPR/Cas9 null mutants of *UCIP2* were generated. A homozygous mutant was selected in the T_2_ generation with an 11 bp deletion of the target 1 sequence (Figure S2). The 11 bp deletion causes a premature stop codon in *UCIP2*. Punch inoculation of the *UCIP2* null mutant showed compromised resistance to *M. oryzae* with larger lesion size and greater relative fungal biomass compared to Nipponbare (Figure 6).

### 2.7 OsUBC26 could work with APIP6 and ubiquitinate AvrPiz‐t

**FIGURE 7:**
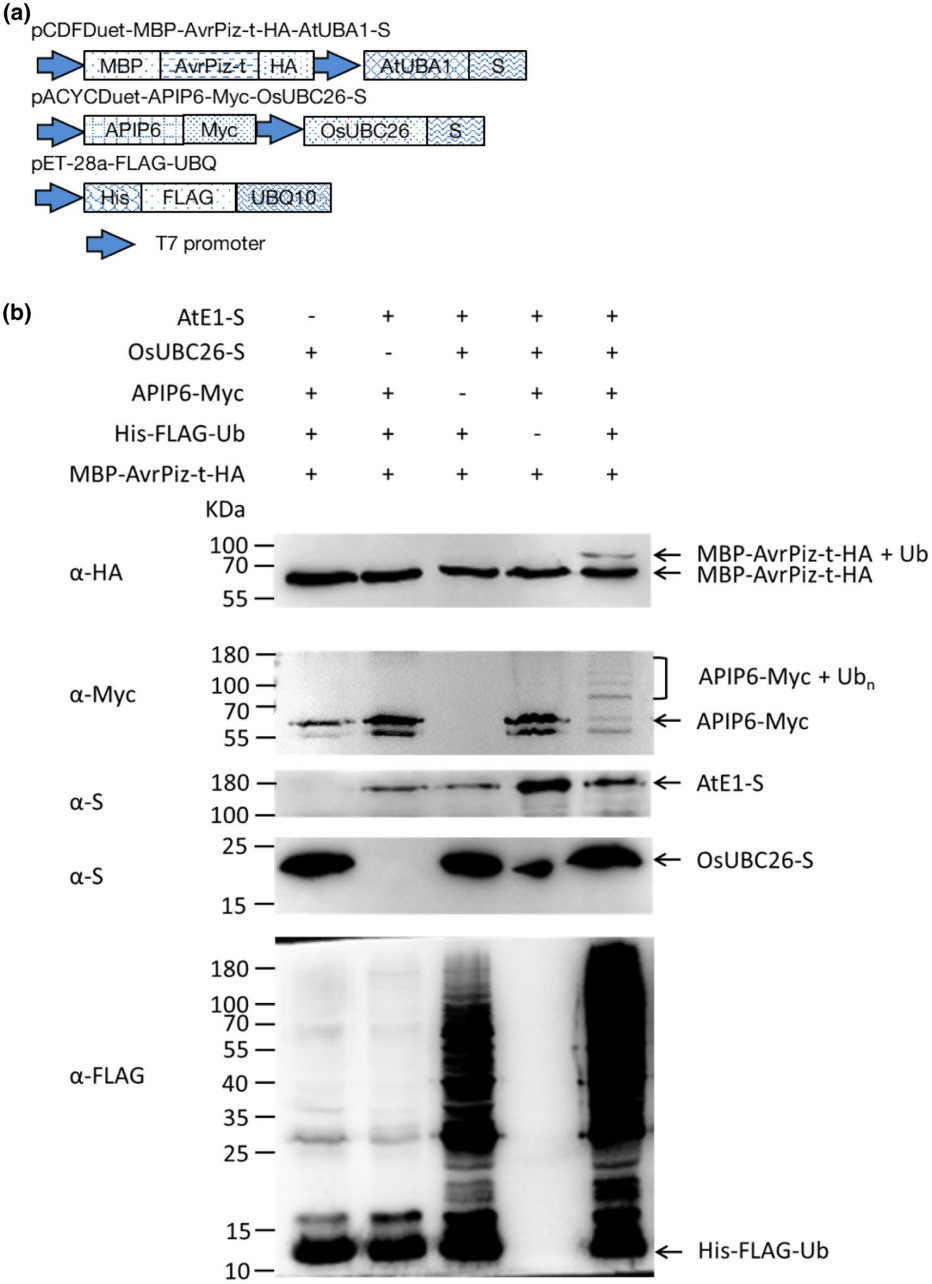
OsUBC26 could work with APIP6 and ubiquitinate AvrPiz‐t in vitro. (a) Schematic representation of the plasmids pCDFDuet‐MBP‐AvrPiz‐t‐HA‐AtUBA1‐S, pACYCDuet‐APIP6‐Myc‐OsUBC26‐S, and pET‐28a‐FLAG‐UBQ. (b) Detection of ubiquitination of AvrPiz‐t. The crude total proteins were separated by sodium dodecyl sulphate polyacrylamide gel electrophoresis and visualized by western blot with corresponding antibodies

During rice–pathogen interaction, E3 ligase activity is widely used by the host to reprogramme the cell to counteract pathogens (Ning et al., 2016). AvrPiz‐t is an avirulence effector from *M. oryzae* that targets a rice E3 ligase APIP6 to suppress PTI. In contrast, APIP6 can ubiquitinate AvrPiz‐t and promote the proteasome degradation of AvrPiz‐t (Park et al., 2012). In an effort to find substrate proteins of UCIP2, we screened the rice cDNA library using UCIP2 as bait and found that APIP6 is a binding partner of UCIP2. An in vitro ubiquitination assay showed that in the presence of Ub, AtE1, OsUBC26, and both APIP6 and UCIP2 were ubiquitinated (Figure S3), which suggests that OsUBC26 may also work with APIP6. We therefore checked whether OsUBC26 could work with APIP6 and ubiquitinate AvrPiz‐t by using a bacterial‐based synthetic approach (Han et al., 2017). This assay demonstrated that ubiquitination of AvrPiz‐t required the presence of AtE1, OsUBC26, APIP6, and ubiquitin (Figure 7). This indicates that OsUBC26 could work with APIP6 and ubiquitinate AvrPiz‐t in vitro.

### 2.8 *OsUBC26* null mutation impairs the proteasome degradation of AvrPiz‐t

**FIGURE 8:**
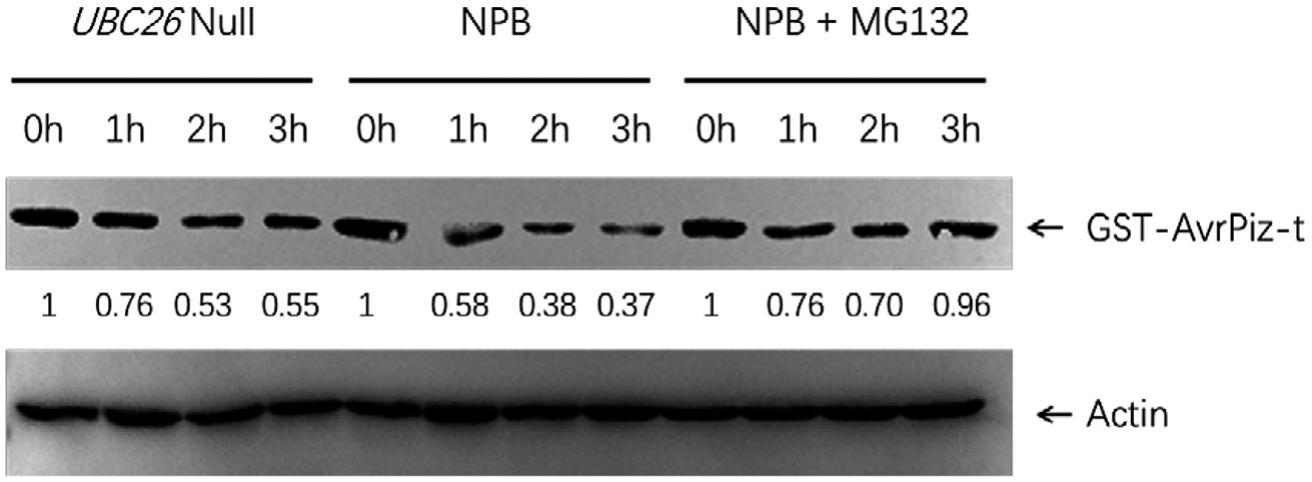
*OsUB26* null mutation impairs proteasome degradation of AvrPiz‐t in rice cells. Total protein was extracted from *OsUBC26* null mutant and Nipponbare with or without MG132. Purified proteins GST‐AvrPiz‐t from *Escherichia coli* BL21 (DE3) were incubated in rice total protein supernatant with the addition of 1 mM ATP and cultured at 28 °C for different times. Actin was used as a loading control. Band intensity was calculated by ImageJ software

As OsUBC26 could work with APIP6 and mediate the ubiquitination of AvrPiz‐t, we wanted to know whether OsUBC26‐APIP6 mediates the degradation of AvrPiz‐t. Cell‐free degradation was conducted and the results showed that the proteasome degradation of AvrPiz‐t was significantly impaired in the *OsUBC26* null mutant group compared to the wild‐type Nipponbare group (Figure 8). When MG132, a specific proteasome inhibitor, was added to the Nipponbare group the degradation of AvrPiz‐t was blocked. This experiment indicated that the degradation of AvrPiz‐t in rice cells was via the 26S proteasome system and OsUBC26 was involved in the ubiquitination and degradation of AvrPiz‐t.

## 3 DISCUSSION

The host ubiquitin proteasome system plays an important role in defence against pathogens. Compared to the deep understanding of the role of ubiquitin E3 ligases in plant immunity, relatively little is known about the role of E2 conjugating enzyme in immunity. In this study, we characterized a ubiquitin‐conjugating enzyme in rice defence. Knockdown and null mutation of *OsUBC26* compromised rice resistance to *M. oryzae*, which indicates that OsUBC26 is essential for rice immunity to this fungus.

Park et al. (2012) found that APIP6 interacts with AvrPiz‐t and promotes the degradation of AvrPiz‐t in *Nicotiana benthamiana* leaves. When they tested the stability of AvrPiz‐t in rice cells, no signal was detected, which suggests that AvrPiz‐t was targeted for degradation in rice cells. However, the degradation of AvrPiz‐t in rice cells via the ubiquitin–26S proteasome system remains uncharacterized. Moreover, which ubiquitin‐conjugating enzyme works with APIP6 and mediates the ubiquitination of AvrPiz‐t in rice cells remains unknown. Here we found that OsUBC26 could work with APIP6 and ubiquitinate AvrPiz‐t in vitro. In addition, *OsUBC26* null mutation impaired the proteasome degradation of AvrPiz‐t in rice cells, which suggests that OsUBC26 may work with APIP6 and promote the ubiquitination and degradation of AvrPiz‐t via the 26S proteasome in rice cells, and help the rice plant to counteract AvrPiz‐t secreted from *M. oryzae*.

In the rice genome there are 48 ubiquitin‐conjugating enzymes and over 1,000 E3 ligases (Bae & Kim, 2014; Zhou et al., 2009). Generally, one ubiquitin‐conjugating enzyme interacts with more than one E3 ligase. Yeast two‐hybrid screening discovered several E3 ligases that interact with OsUBC26 (Table 1). At least four RING‐type E3 ligases, UCIP1, UCIP2, UCIP3, and UCIP4, interact with OsUBC26. We further confirmed the interaction between OsUBC26 and UCIP2 in yeast using the full‐length coding sequence of UCIP2 and then GST pull‐down assay. Our work also revealed that OsUBC26 could work with APIP6 to ubiquitinate AvrPiz‐t in vitro. Bae and Kim (2014) characterized the interaction modes of rice E2s with ARM‐U‐box E3 ligases by yeast two‐hybrid assays and showed that OsUBC26 also interacts with SPL11, OsPUB33, OsPUB36, OsPUB38, and OsPUB41. Hence, OsUBC26 may interact with other E3 ligases in addition to UCIP2.

In addition to E3 ligases, there were some interesting additional candidate binding partners of OsUBC26 identified in the library screening. UCIP7, a *cis*‐zeatin *O*‐glucosyltransferase, converts *cis*‐zeatin to its inactive form. *Cis*‐zeatin functions as a kind of cytokinin, which indicates that OsUBC26 may have interplay with cytokinin signalling and regulate cytokinin activity by *O*‐glucosylation. UCIP9, a thioesterase domain‐containing protein, may play a role in the ubiquitin binding status of OsUBC26 as ubiquitin is bound to E2 through a thioester bond. UCIP6 and UCIP10 are kinase domain‐containing proteins, which indicates that the activity of OsUBC26 may be regulated by phosphorylation. UCIP5 and UCIP17 are transcription factors, which suggests that OsUBC26 may regulate UCIP5 and UCIP17 transcriptional activity by directly interacting with them.

Our RT‐qPCR results showed that the expression of *OsUBC26* was induced by *M. oryzae* inoculation and Me‐JA treatment but not SA. This may imply that the JA pathway is involved in *OsUBC26*. However, null mutation of *OsUBC26* lowered the expression of *WRKY45*, and WRKY45 is a transcription factor involved in rice resistance via the SA signalling pathway that is regulated by the ubiquitin proteasome system (Matsushita et al., 2013). With regard to the high innate level of SA in rice plants, both JA and SA signalling pathways may be involved in OsUBC26‐mediated rice immunity.

The results of Zhong et al. (2018) indicated that OsDjA6 is a negative regulator of *WRKY45* expression, and OsZFP1 (UCIP2) was discovered as a working partner of OsDjA6. However, the expression of *WRKY45* in *UCIP2* null mutant shows no significant difference compared to wild‐type plants. OsUBC26‐UCIP2 did not ubiquitinate OsDjA6 in vitro (data not shown) so the regulation of *WRKY45* expression by OsUBC26 may be a separate way. Further study is required to uncover how OsUBC26 and OsDjA6 regulate the expression of *WRKY45*.

## 4 EXPERIMENTAL PROCEDURES

### 4.1 Plant materials and growth conditions

Rice seeds were soaked into water for 48 hr at room temperature and the water was changed every 12 hr. The seeds were pregerminated in an incubator at 37 °C for 1 day. After germination, the seeds were planted in small pots and kept in a greenhouse at 28 °C light/23 °C dark and 70% relative humidity under a photoperiod of 14 hr light/ 10 hr dark for growth.

### 4.2 Spraying inoculation and Me‐JA treatment

*M. oryzae* isolate Guy11 was pregrown on rice bran medium for 10 days in the dark. The aerial hyphae were flattened using a sterilized glass slide and the growth plates were incubated under light to encourage a uniform crop of conidia. The concentration of conidia was adjusted to 1–2 × 10^5^ spores/ml with 0.02% Tween‐20. Three‐week‐old rice seedlings were used for spray inoculation. After spraying, rice seedlings were kept in dark and high humidity conditions for 24 hr. The seedlings were then moved to a solar greenhouse with high humidity and grown for 7 days. ImageJ software was used to quantify the lesion area. For Me‐JA treatment, 3‐week‐old rice seedlings were sprayed with 100 µM Me‐JA, then kept in an incubator at 28 °C with high humidity. Samples were taken at time points of 0, 1, 3, 6, 12, and 24 hpt for RT‐qPCR analysis.

### 4.3 Punch inoculation and disease resistance evaluations

For punch inoculation, *M. oryzae* isolate FJ86‐CT was used. The concentration of the fungal spores was adjusted to 5 × 10^5^ spores/ml with 0.02% Tween‐20 and 7 µl of spore suspension was used for punch inoculation as described by Zhong et al. (2018). After punch inoculation, the seedlings were kept in a growth chamber at 28 °C light/25 °C dark and 90% relative humidity under a photoperiod of 12 hr light/12 hr dark. After 7–9 days’ growth, the diseased leaves were sampled for relative fungal biomass quantitation using the DNA‐based quantitative PCR method as described by Park et al. (2012).

### 4.4 RNA isolation and RT‐qPCR

RNA was extracted by using a total RNA extraction kit (Promega) following the manufacturer's instructions. The RNA concentration was determined by NanoDrop 2000 machine (Eppendorf). A PrimeScript RT‐PCR Kit (Takara) was used for reverse transcription and TB Green Premix Ex Taq (Takara) was used for qPCR analysis following the manufacturer's instructions. Primers are shown in Table S3.

### 4.5 RNAi

For building of the *OsUBC26* RNAi line, the pTCK‐303 plasmid and primer pair UBC26‐RNAi‐F/UBC26‐RNAi‐R was used. Sense sequence ligation using *Spe*I/*Sac*I enzyme digestion sites and subsequent *Kpn*I/*Bam*HI enzyme digestion sites was used to obtain an insert for antisense sequence ligation. *Agrobacterium tumefaciens* LBA4404 was used for the transformation of rice cultivar Kitaake callus. All the transgenic seedlings were confirmed by PCR and β‐glucuronidase (GUS) staining analysis. The T_2_ generation of RNAi lines was used for the RT‐qPCR and inoculation experiments.

### 4.6 Gene editing

To generate the gene null transgenic plants, two targets were designed using the CRISPR‐GE toolkit (Xie et al., 2017). The two targets were ligated to promoters and inserted into the pYLCRISPR/*Cas9*‐MTmono vector (Ma et al., 2015). *A*. *tumefaciens* EHA105 was used for the transformation of rice cultivar Nipponbare callus. The gene editing result was further checked by sequencing of the amplified targeted sequence. Homozygotes were selected for phenotypic analysis.

### 4.7 E2 charging assay

For the E2 activity assay, pET28a‐ubiquitin, pET28a‐AtE1, and pET28a‐OsUBC26 were expressed in *Escherichia coli* BL21 (DE3) and purified using Ni‐NTA agarose (Qiagen) separately. The E2 charging assay was conducted according to protocols described elsewhere (Zhao et al., 2012). The final result was visualized using anti‐ubiquitin antibody.

### 4.8 Yeast two‐hybrid screening

Coding region sequences of *OsUBC26* and *UCIP2* were constructed in pDBLeu vector as bait. 3‐aminotriazole (3AT) sensitivity was determined by plate yeast transformants containing bait plasmid and empty prey plasmid onto SC−Leu−Trp−His plates containing 3AT at concentrations of 0, 10, 25, 50, 75, and 100 mM. *Saccharomyces cerevisiae* MaV203 (Invitrogen) containing bait plasmid was used for making competent cells. The cDNA library was transformed into competent cells and plated onto SC−Leu−Trp−His plus 25 mM 3AT plates to select positive clones.

### 4.9 GST pull‐down

For the GST pull‐down assay, the coding sequence of *OsUBC26* was cloned into pGEX‐KG by using *Bam*HI and *Eco*RI restriction sites. The open reading frame of *UCIP2* was inserted into pMAL‐c5X by *Not*I and *Eco*RI restriction sites. Both constructions were expressed in *E*. *coli* BL21 (DE3). The ultrasonic lysate containing GST:OsUBC26 was bound to glutathione magarose beads overnight at 4 °C using GST binding buffer (140 mM NaCl, 2.7 mM KCl, 10 mM Na_2_HPO_4_, 1.8 mM KH_2_PO_4_, pH 7.4). MBP binding buffer (20 mM Tris‐HCl, 200 mM NaCl, 1 mM EDTA, pH 7.4) was used for MBP:UCIP2 ultrasonic lysis. Beads were washed three times with GST binding buffer then MBP:UCIP2 ultrasonic lysate was added to the system and incubated 8–12 hr at 4 °C. Input and pull‐down proteins were visualized by sodium dodecyl sulphate polyacrylamide gel electrophoresis (SDS‐PAGE) and western blot analysis with corresponding antibodies.

### 4.10 In vitro ubiquitination assays

Plasmids pCDFDuet‐MBP‐ABI3‐HA‐AtUBA1‐S, pACYCDuet‐AIP2‐Myc‐UBC8‐S, and pET‐28a‐FLAG‐UBQ were built by Han et al. (2017). We replaced *AIP2* in the plasmid of pACYCDuet‐AIP2‐Myc‐UBC8‐S by *APIP6* using *Bam*HI and *Stu*I restriction sites. Plasmids pCDFDuet‐MBP‐UCIP2‐HA‐AtUBA1‐S, pCDFDuet‐MBP‐AvrPiz‐t‐HA‐AtUBA1‐S, and pACYCDuet‐APIP6‐Myc‐OsUBC26‐S were constructed using the recombination‐type‐cloning method (ClonExpress II One Step Cloning Kit; Vazyme). For the deletion of *AtUBA1* in the plasmid pCDFDuet‐MBP‐AvrPiz‐t‐HA‐AtUBA1‐S and deletion of *OsUBC26* in the plasmid of pACYCDuet‐APIP6‐Myc‐OsUBC26‐S, PCR‐based amplification was used with specific restriction enzyme sites *Cla*I and *Spe*I, respectively. For the deletion of *APIP6*, pACYCDuet‐APIP6‐Myc‐OsUBC26‐S was digested by *Bam*HI and *Stu*I, and then blunt‐ended by T4 DNA polymerase (Takara).

### 4.11 Cell‐free degradation

Cell‐free degradation was performed as described by Kong et al. (2015). Briefly, total protein from 3‐week‐old rice seedlings was extracted with native extraction buffer (50 mM Tris‐MES, pH 8.0, 0.5 M sucrose, 1 mM MgCl_2_, 10 mM EDTA, pH 8.0, 5 mM dithiothreitol [DTT], and protease inhibitor cocktail) with or without 100 µM MG132. Insoluble debris was pelleted twice by centrifugation at 13,000 rpm for 10 min at 4 °C. For construction of GST‐AvrPiz‐t plasmid, pGEX‐KG was digested with *Sma*I and *Sal*I, and *AvrPiz‐t* coding sequence was inserted in it using the recombination‐type‐cloning method (ClonExpress II One Step Cloning Kit; Vazyme). Purified proteins GST‐AvrPiz‐t from *E. coli* BL21 (DE3) were incubated in rice total protein supernatant with addition of 1 mM ATP and cultured at 28 °C for different times. Anti‐GST antibody was used to detect the GST‐AvrPiz‐t protein level by immunoblotting analysis.

## Supporting information


**FIGURE S1** Genome editing events occurring in *OsUBC26* null mutants

**FIGURE S2** Genome editing events occurring in *UCIP2* null mutants

**FIGURE S3** Both APIP6 and UCIP2 were ubiquitinated when coexpressed in *Escherichia coli*

**TABLE S1** Up‐regulated genes in *OsUBC26* null mutant

**TABLE S2** Down‐regulated genes in *OsUBC26* null mutant

**TABLE S3** Primers used in this study

## Data Availability

The data that support the findings of this study are available from the corresponding author upon reasonable request.

## References

[mpp13132-bib-0001] Bae, H. & Kim, W.T. (2014) Classification and interaction modes of 40 rice E2 ubiquitin‐conjugating enzymes with 17 rice ARM‐U‐box E3 ubiquitin ligases. Biochemical and Biophysical Research Communications, 444, 575–580. 24486490 10.1016/j.bbrc.2014.01.098

[mpp13132-bib-0002] Chung, E. , Cho, C.W. , So, H.A. , Kang, J.S. , Chung, Y.S. & Lee, J.H. (2013) Overexpression of *VrUBC1*, a mung bean E2 ubiquitin‐conjugating enzyme, enhances osmotic stress tolerance in *Arabidopsis* . PLoS One, 8, e66056. 23824688 10.1371/journal.pone.0066056 PMC3688854

[mpp13132-bib-0003] Dielen, A.S. , Badaoui, S. , Candresse, T. & German‐Retana, S. (2010) The ubiquitin/26S proteasome system in plant–pathogen interactions: a never‐ending hide‐and‐seek game. Molecular Plant Pathology, 11, 293–308. 20447278 10.1111/j.1364-3703.2009.00596.x PMC6640532

[mpp13132-bib-0004] Han, Y. , Sun, J. , Yang, J. , Tan, Z. , Luo, J. & Lu, D. (2017) Reconstitution of the plant ubiquitination cascade in bacteria using a synthetic biology approach. The Plant Journal, 91, 766–776. 28509348 10.1111/tpj.13603

[mpp13132-bib-0005] Hershko, A. (2005) The ubiquitin system for protein degradation and some of its roles in the control of the cell division cycle. Cell Death and Differentiation, 12, 1191–1197. 16094395 10.1038/sj.cdd.4401702

[mpp13132-bib-0006] Jeon, E.H. , Pak, J.H. , Kim, M.J. , Kim, H.J. , Shin, S.H. , Lee, J.H. et al. (2012) Ectopic expression of ubiquitin‐conjugating enzyme gene from wild rice, *OgUBC1*, confers resistance against UV‐B radiation and *Botrytis* infection in *Arabidopsis thaliana* . Biochemical and Biophysical Research Communications, 427, 309–314. 23000158 10.1016/j.bbrc.2012.09.048

[mpp13132-bib-0007] Kong, L. , Cheng, J. , Zhu, Y. , Ding, Y. , Meng, J. , Chen, Z. et al. (2015) Degradation of the ABA co‐receptor ABI1 by PUB12/13 U‐box E3 ligases. Nature Communications, 6, 8630. 10.1038/ncomms9630 PMC4667695 26482222

[mpp13132-bib-0008] Kraft, E. , Stone, S.L. , Ma, L. , Su, N. , Gao, Y. , Lau, O.‐S. et al. (2005) Genome analysis and functional characterization of the E2 and RING‐type E3 ligase ubiquitination enzymes of *Arabidopsis* . Plant Physiology, 139, 1597–1611. 16339806 10.1104/pp.105.067983 PMC1310545

[mpp13132-bib-0009] Ma, X. , Zhang, Q. , Zhu, Q. , Liu, W. , Chen, Y. , Qiu, R. et al. (2015) A robust CRISPR/Cas9 system for convenient, high‐efficiency multiplex genome editing in monocot and dicot plants. Molecular Plant, 8, 1274–1284. 25917172 10.1016/j.molp.2015.04.007

[mpp13132-bib-0010] Marino, D. , Peeters, N. & Rivas, S. (2012) Ubiquitination during plant immune signaling. Plant Physiology, 160, 15–27. 22689893 10.1104/pp.112.199281 PMC3440193

[mpp13132-bib-0011] Matsushita, A. , Inoue, H. , Goto, S. , Nakayama, A. , Sugano, S. , Hayashi, N. et al. (2013) Nuclear ubiquitin proteasome degradation affects WRKY45 function in the rice defense program. The Plant Journal, 73, 302–313. 23013464 10.1111/tpj.12035 PMC3558880

[mpp13132-bib-0012] Millyard, L. , Lee, J. , Zhang, C. , Yates, G. & Sadanandom, A. (2016) The ubiquitin conjugating enzyme, TaU4 regulates wheat defence against the phytopathogen *Zymoseptoria tritici* . Scientific Reports, 6, 35683. 27759089 10.1038/srep35683 PMC5069635

[mpp13132-bib-0013] Ning, Y. , Wang, R. , Shi, X. , Zhou, X. & Wang, G.L. (2016) A layered defense strategy mediated by rice E3 ubiquitin ligases against diverse pathogens. Molecular Plant, 9, 1096–1098. 27381441 10.1016/j.molp.2016.06.015

[mpp13132-bib-0014] Ono, E. , Wong, H.L. , Kawasaki, T. , Hasegawa, M. , Kodama, O. & Shimamoto, K. (2001) Essential role of the small GTPase Rac in disease resistance of rice. Proceedings of the National Academy of Sciences USA, 98, 759–764. 10.1073/pnas.021273498 PMC14661 11149940

[mpp13132-bib-0015] Park, C.H. , Chen, S. , Shirsekar, G. , Zhou, B. , Khang, C.H. , Songkumarn, P. et al. (2012) The *Magnaporthe oryzae* effector AvrPiz‐t targets the RING E3 Ubiquitin Ligase APIP6 to suppress pathogen‐associated molecular pattern‐triggered immunity in rice. The Plant Cell, 24, 4748–4762. 23204406 10.1105/tpc.112.105429 PMC3531864

[mpp13132-bib-0016] Park, C.H. , Shirsekar, G. , Bellizzi, M. , Chen, S. , Songkumarn, P. , Xie, X. et al. (2016) The E3 ligase APIP10 connects the effector AvrPiz‐t to the NLR receptor Piz‐t in rice. PLoS Pathogens, 12, e1005529. 27031246 10.1371/journal.ppat.1005529 PMC4816579

[mpp13132-bib-0017] Shimono, M. , Koga, H. , Akagi, A. , Hayashi, N. , Goto, S. , Sawada, M. et al. (2012) Rice WRKY45 plays important roles in fungal and bacterial disease resistance. Molecular Plant Pathology, 13, 83–94. 21726399 10.1111/j.1364-3703.2011.00732.x PMC6638719

[mpp13132-bib-0018] Shimono, M. , Sugano, S. , Nakayama, A. , Jiang, C.‐J. , Ono, K. , Toki, S. et al. (2007) Rice WRKY45 plays a crucial role in benzothiadiazole‐inducible blast resistance. The Plant Cell, 19, 2064–2076. 17601827 10.1105/tpc.106.046250 PMC1955718

[mpp13132-bib-0019] Smalle, J. & Vierstra, R.D. (2004) The ubiquitin 26S proteasome proteolytic pathway. Annual Review of Plant Biology, 55, 555–590. 10.1146/annurev.arplant.55.031903.141801 15377232

[mpp13132-bib-0020] Stone, S.L. (2014) The role of ubiquitin and the 26S proteasome in plant abiotic stress signaling. Frontiers in Plant Science, 5, 135. 24795732 10.3389/fpls.2014.00135 PMC3997020

[mpp13132-bib-0021] Varshavsky, A. (1997) The ubiquitin system. Trends in Biochemical Sciences, 22, 383–387. 9357313 10.1016/s0968-0004(97)01122-5

[mpp13132-bib-0022] Wan, X. , Mo, A. , Liu, S. , Yang, L. & Li, L. (2011) Constitutive expression of a peanut ubiquitin‐conjugating enzyme gene in *Arabidopsis* confers improved water‐stress tolerance through regulation of stress‐responsive gene expression. Journal of Bioscience and Bioengineering, 111, 478–484. 21193345 10.1016/j.jbiosc.2010.11.021

[mpp13132-bib-0023] Wang, Y. , Pi, L. , Chen, X. , Chakrabarty, P.K. , Jiang, J. , De Leon, A.L. et al. (2006) Rice XA21 binding protein 3 is a ubiquitin ligase required for full *Xa21*‐mediated disease resistance. The Plant Cell, 18, 3635–3646. 17172358 10.1105/tpc.106.046730 PMC1785399

[mpp13132-bib-0024] Wenzel, D. , Stoll, K. & Klevit, R. (2011) E2s: structurally economical and functionally replete. The Biochemical Journal, 433, 31–42. 21158740 10.1042/BJ20100985 PMC3118098

[mpp13132-bib-0025] Xie, X. , Ma, X. , Zhu, Q. , Zeng, D. , Li, G. & Liu, Y.G. (2017) CRISPR‐GE: A convenient software toolkit for CRISPR‐based genome editing. Molecular Plant, 10, 1246–1249. 28624544 10.1016/j.molp.2017.06.004

[mpp13132-bib-0026] Ye, Y. & Rape, M. (2009) Building ubiquitin chains: E2 enzymes at work. Nature Reviews Molecular Cell Biology, 10, 755–764. 19851334 10.1038/nrm2780 PMC3107738

[mpp13132-bib-0027] Zeng, L. , Qu, S. , Bordeos, A. , Yang, C. , Baraoidan, M. , Yan, H. et al. (2004) Spotted leaf11, a negative regulator of plant cell death and defense, encodes a U‐box/armadillo repeat protein endowed with E3 ubiquitin ligase activity. The Plant Cell, 16, 2795–2808. 15377756 10.1105/tpc.104.025171 PMC520972

[mpp13132-bib-0028] Zeng, L.R. , Vega‐Sanchez, M.E. , Zhu, T. & Wang, G.L. (2006) Ubiquitination‐mediated protein degradation and modification: an emerging theme in plant–microbe interactions. Cell Research, 16, 413–426. 16699537 10.1038/sj.cr.7310053

[mpp13132-bib-0029] Zhao, C.‐J. , Wang, A.‐R. , Shi, Y.‐J. , Wang, L.‐Q. , Liu, W.‐D. , Wang, Z.‐H. et al. (2008) Identification of defense‐related genes in rice responding to challenge by *Rhizoctonia solani* . Theoretical and Applied Genetics, 116, 501–516. 18075727 10.1007/s00122-007-0686-y

[mpp13132-bib-0030] Zhao, Q. , Liu, L. & Xie, Q. (2012) In vitro protein ubiquitination assay. Plant Signalling Networks, Methods in Molecular Biology. 876, Germany: Springer. pp.163–172. 10.1007/978-1-61779-809-2_13 22576094

[mpp13132-bib-0031] Zhong, X. , Yang, J. , Shi, Y. , Wang, X. & Wang, G.L. (2018) The DnaJ protein OsDjA6 negatively regulates rice innate immunity to the blast fungus *Magnaporthe oryzae* . Molecular Plant Pathology, 19, 607–614. 28220688 10.1111/mpp.12546 PMC6638105

[mpp13132-bib-0032] Zhou, B. , Mural, R.V. , Chen, X. , Oates, M.E. , Connor, R.A. , Martin, G.B. et al. (2017) A subset of ubiquitin‐conjugating enzymes is essential for plant immunity. Plant Physiology, 173, 1371–1390. 27909045 10.1104/pp.16.01190 PMC5291023

[mpp13132-bib-0033] Zhou, D. , Zhou, X. , Li, L. & Su, Z. (2009) PlantsUPS: a database of plants' ubiquitin proteasome system. BMC Genomics, 10, 227. 19445698 10.1186/1471-2164-10-227 PMC2690602

[mpp13132-bib-0034] Zhou, G.A. , Chang, R.Z. & Qiu, L.J. (2010) Overexpression of soybean ubiquitin‐conjugating enzyme gene *GmUBC2* confers enhanced drought and salt tolerance through modulating abiotic stress‐responsive gene expression in *Arabidopsis* . Plant Molecular Biology, 72, 357–367. 19941154 10.1007/s11103-009-9575-x PMC2816239

