# Supplementary material for: Rice ubiquitin‐conjugating enzyme OsUBC26 is essential for immunity to the blast fungus Magnaporthe oryzae
Source: Mol Plant Pathol. 2021 Aug 30;22(12):1613–23. doi: 10.1111/mpp.13132 (PMC8578843; doi:10.1111/mpp.13132)
Supplement: Supplementary file 1 — FIGURE S1 Genome editing events occurring in OsUBC26 null mutants [file MPP-22-1613-s004.docx]

YH28-8 Target 1


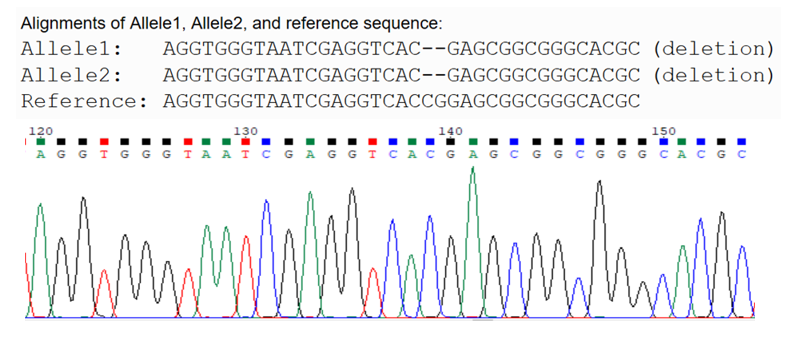


YH28-8 Target 2


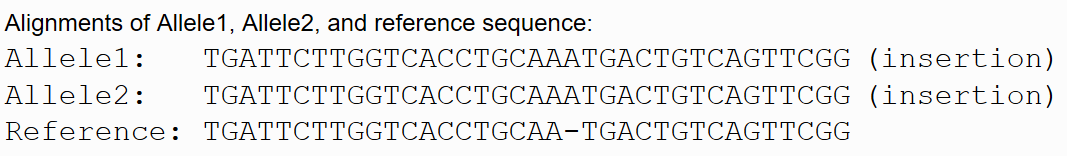


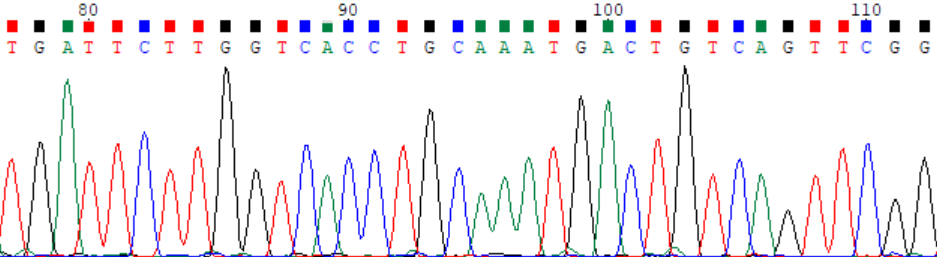


YH34-9 Target 1
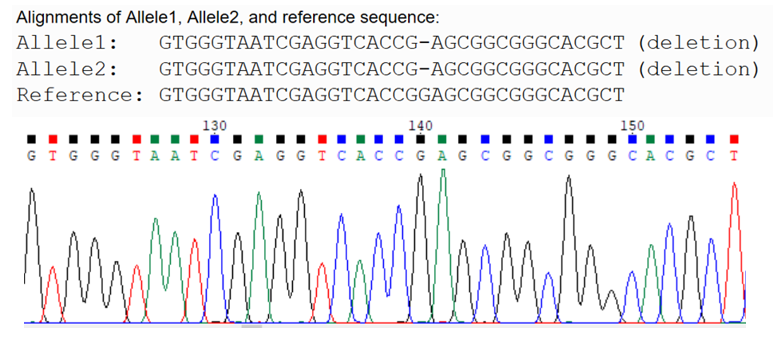


YH34-9 Target 2

Alignments of Allele1, Allele2, and reference sequence

Allele1: TGATTCTTGGTCACCTGCAATGACTGTCAGTTCGG(wild type)

Allele2: TGATTCTTGGTCACCTGCAATGACTGTCAGTTCGG(wild type)

Reference: TGATTCTTGGTCACCTGCAATGACTGTCAGTTCGG


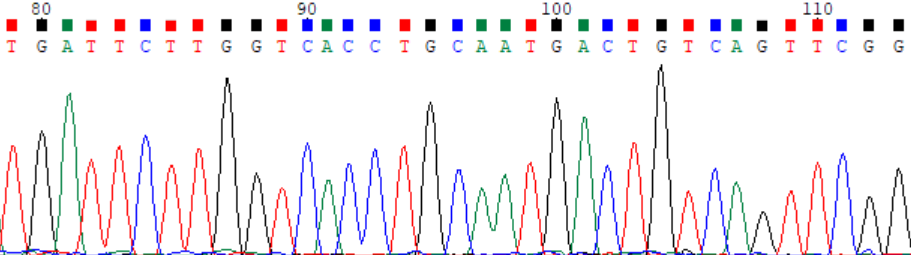


Fig. S1. Genome editing events occurred in *OsUBC26* null mutants
