# Supplementary figures and images for: Rice ubiquitin‐conjugating enzyme OsUBC26 is essential for immunity to the blast fungus Magnaporthe oryzae
Source: Mol Plant Pathol. 2021 Aug 30;22(12):1613–23. doi: 10.1111/mpp.13132 (PMC8578843; doi:10.1111/mpp.13132)

*UCIP2* KO Target 1


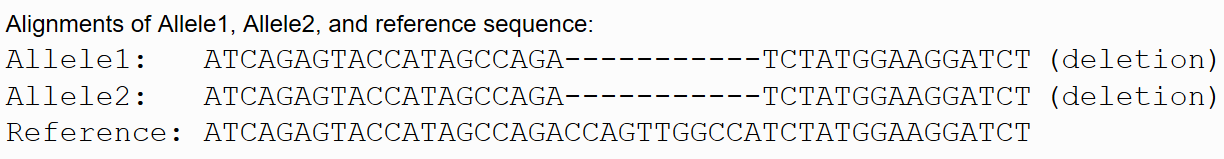


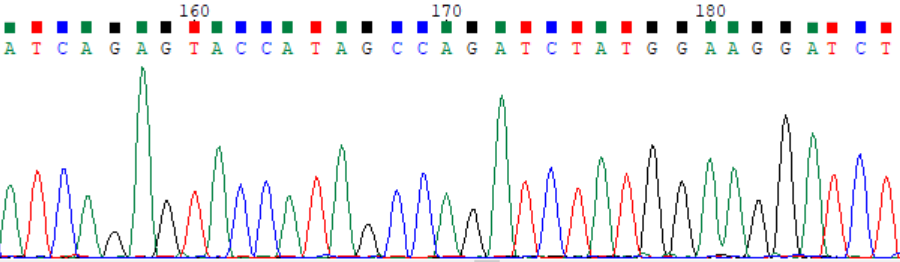


*UCIP2* KO Target 2


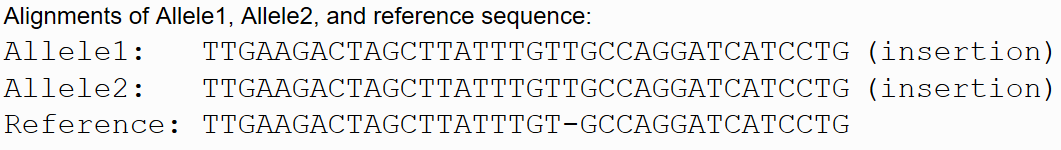


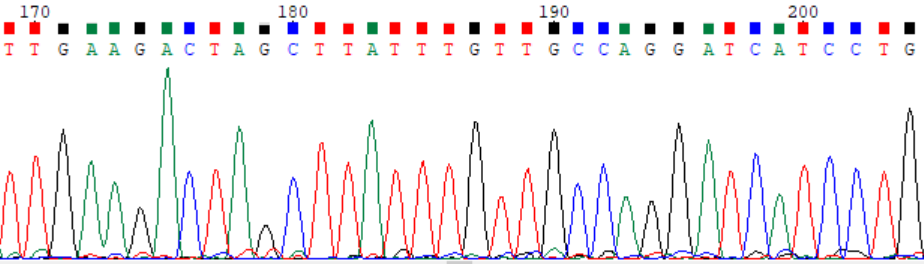


Fig. S2. Genome editing events occurred in *UCIP2* null mutants

Supplement: Supplementary file 2 — FIGURE S2 Genome editing events occurring in UCIP2 null mutants [file MPP-22-1613-s001.docx]
