# Supplementary material for: Rice ubiquitin‐conjugating enzyme OsUBC26 is essential for immunity to the blast fungus Magnaporthe oryzae
Source: Mol Plant Pathol. 2021 Aug 30;22(12):1613–23. doi: 10.1111/mpp.13132 (PMC8578843; doi:10.1111/mpp.13132)
Supplement: Supplementary file 3 — FIGURE S3 Both APIP6 and UCIP2 were ubiquitinated when coexpressed in Escherichia coli [file MPP-22-1613-s003.docx]

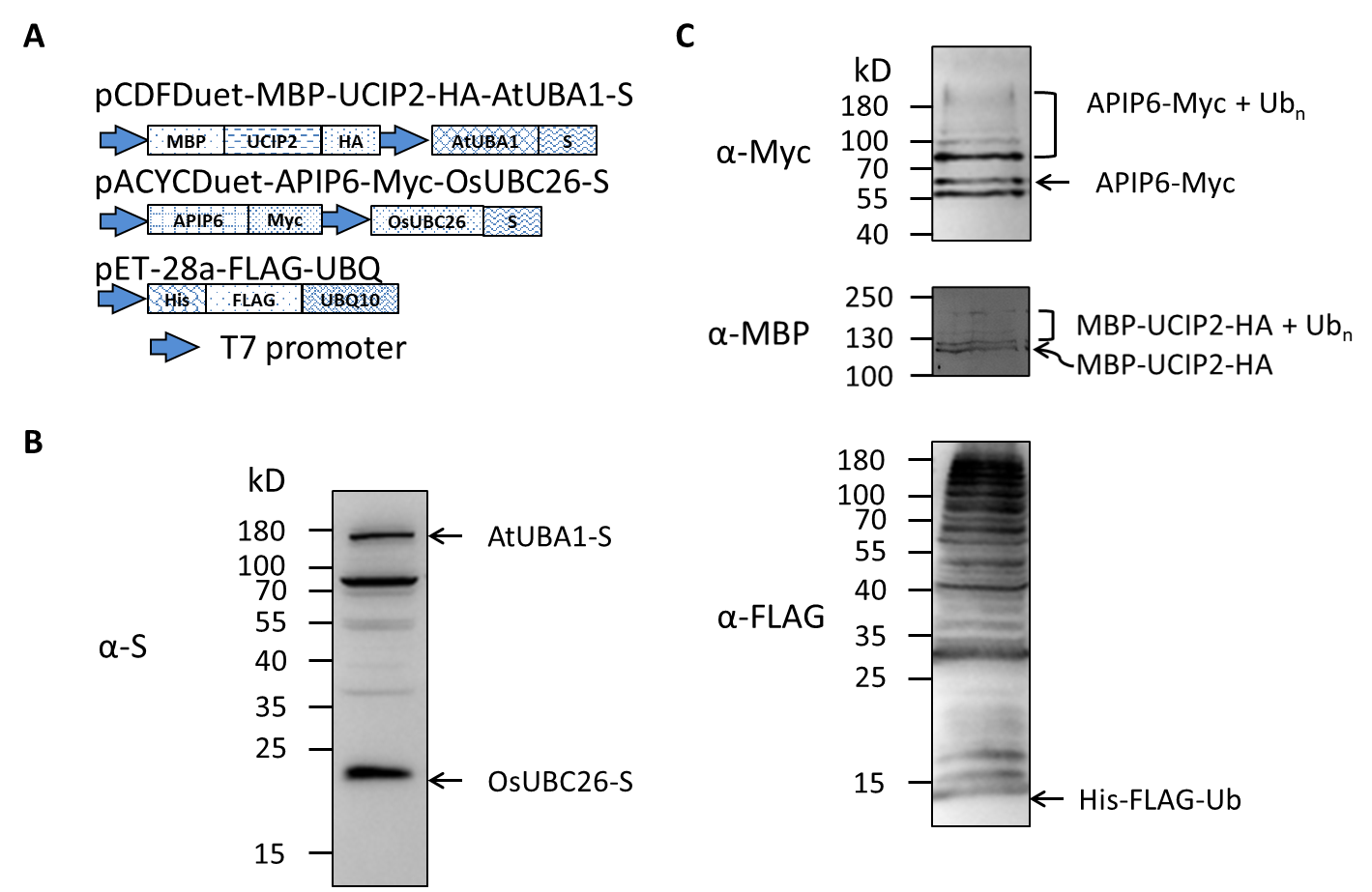


Fig. S3. Both APIP6 and UCIP2 were ubiquitinated when co-expressed in *E. coli*

(A)Schematic representation of the plasmids pCDFDuet-MBP-UCIP2-HA-AtUBA1-S, pACYCDuet-APIP6-Myc-OsUBC26-S and pET-28a-FLAG-UBQ. (B) Detection of AtUBA-S and OsUBC26-S by anti-S antibody. (C) Detection of ubiquitination of APIP6 and UCIP2 by corresponding antibodies.
