# Supplementary material for: Rice ubiquitin‐conjugating enzyme OsUBC26 is essential for immunity to the blast fungus Magnaporthe oryzae
Source: Mol Plant Pathol. 2021 Aug 30;22(12):1613–23. doi: 10.1111/mpp.13132 (PMC8578843; doi:10.1111/mpp.13132)
Supplement: Supplementary file 6 — TABLE S3 Primers used in this study [file MPP-22-1613-s002.docx]

Table S3. Primers used in this study

| Primer Name | Sequences(5’-3’) | Usage |
| --- | --- | --- |
| UBC26-KO-T1-F | GCCGGTAATCGAGGTCACCGGAG | Gene knockout |
| UBC26-KO-T1-R | AAACCTCCGGTGACCTCGATTAC | Gene knockout |
| UBC26-KO-T2-F | GGCACCGAACTGACAGTCATTGC | Gene knockout |
| UBC26-KO-T2-R | AAACGCAATGACTGTCAGTTCGG | Gene knockout |
| UCIP2-KO-T1-F | GGCAGTACCATAGCCAGACCAGT | Gene knockout |
| UCIP2-KO-T1-R | AAACACTGGTCTGGCTATGGTAC | Gene knockout |
| UCIP2-KO-T2-F | GGCAAGACTAGCTTATTTGTGCC | Gene knockout |
| UCIP2-KO-T2-R | AAACGGCACAAATAAGCTAGTCT | Gene knockout |
| U-F | CTCCGTTTTACCTGTGGAATCG | Gene knockout |
| gRNA-R | CGGAGGAAAATTCCATCCAC | Gene knockout |
| Uctcg-B1’ | TTCAGAGGTCTCTCTCGCACTGGAATCGGCAGCAAAGG | Gene knockout |
| gRctga-B2 | AGCGTGGGTCTCGTCAGGGTCCATCCACTCCAAGCTC | Gene knockout |
| Uctga-B2’ | TTCAGAGGTCTCTCTGACACTGGAATCGGCAGCAAAGG | Gene knockout |
| gRcggt-BL | AGCGTGGGTCTCGACCGGGTCCATCCACTCCAAGCTC | Gene knockout |
| UBC26-T1-F | GATTTCTTGTTTCTTCTCCTGTGG | Gene knockout |
| UBC26-T1-R | AACAGATGAGAAGAAACGAAAGGAT | Gene knockout |
| UBC26-T1-seq | TCTCCTGTGGATGAACCG | Gene knockout |
| UBC26-T2-F | TAGAAACAGGACTACTTCTTGATCC | Gene knockout |
| UBC26-T2-R | AAACACCACATTATAGCAAGATACC | Gene knockout |
| UBC26-T2-seq | GATTGTGAACCAAAAGAG | Gene knockout |
| UCIP2-T1-F | AGTTGTTCTAGGAAGCATGGATG | Gene knockout |
| UCIP2-T1-R | TTGACATATTCCCACTGTGGTATTC | Gene knockout |
| UCIP2-T1-seq | AATGCTGGTGAAAGTGCT | Gene knockout |
| UCIP2-T2-F | ATGAGCATAGAGATATGCGGCT | Gene knockout |
| UCIP2-T2-R | TATAACACTAGCCAATTCCACACTG | Gene knockout |
| UCIP2-T2-seq | GAGATATGCGGCTAGATA | Gene knockout |
| OsUG-F | TTCTGGTCCTTCCACTTTCAG | qRT PCR |
| OsUG-R | ACGATTGATTTAACCAGTCCATGA | qRT PCR |
| MoPot2-F | ACGACCCGTCTTTACTTATTTGG | qRT PCR |
| MoPot2-R | AAGTAGCGTTGGTTTTGTTGGAT | qRT PCR |
| UBC26-qF | GTTCGGTCTGCATCAGCATTTTG | qRT PCR |
| UBC26-qR | CCATCTGGTCTCCTTCGGCG | qRT PCR |
| WRKY45-qF | CGGGTAAAACGATCGAAAGA | qRT PCR |
| WRKY45-qR | TTTCGAAAGCGGAAGAACAG | qRT PCR |
| Actin-F | GAGTATGATGAGTCGGGTCCAG | qRT PCR |
| Actin-R | ACACCAACAATCCCAAACAGAG | qRT PCR |
| UBC26-EcoRI-F | CGCTTAAGATGACGAGCTCCTCATC | E2 activity |
| UBC26-HindIII-R | CCCTTCGAACTTACACTTTGTCATCATG | E2 activity |
| UBC26-SmaI-F | TCCCCCGGGTATGACGAGCTCCTCATCTCCTTC | Yeast two hybrid |
| UBC26-NotI-R | ATAAGAATGCGGCCGCTTACACTTTGTCATCATGGAACCAC | Yeast two hybrid |
| UBC26-BamHI-F | CGGGATCCATGACGAGCTCCTCATCTCCTTC | GST Pull-down |
| UBC26-EcoRI-R | CCGGAATTCTTACACTTTGTCATCATGGAAC | GST Pull-down |
| UCIP2- NotI-F | ATAAGAATGCGGCCGCATGGCATATAGAAATACGGTATGCACTCC | GST Pull-down |
| UCIP2- EcoRI-R | CGGAATTCTTATCCACTTCTTCTTCCGGTTGAC | GST Pull-down |
| UCIP2-F | ATGGCATATAGAAATACGGTATGCACTCC | Clone |
| UCIP2-R | TTATCCACTTCTTCTTCCGGTTGAC | Clone |
| UCIP2-SmaI-F | TCCCCCGGGAATGGCATATAGAAATACGGTATGCACTCC | Yeast two hybrid |
| UCIP2-NotI-R | ATAAGAATGCGGCCGCATTATCCACTTCTTCTTCCGGTTGAC | Yeast two hybrid |
| BamH1-APIP6-F | CGCGGATCCATGGGTGCGAGGGAGGAGGTG | Replace AIP2 |
| APIP6-Stu1-R | AAAAGGCCTCATCCTTGGGGTGTGCATTTGC | Replace AIP2 |
| UBC26-Insert-F | CAGATCTCAATTGGATATCGATGACGAGCTCCTCATCTCCTTC | Replace UBC8 |
| UBC26-Insert-R | GTTTCTTTACCAGACTCGAGCACTTTGTCATCATGGAACCACC | Replace UBC8 |
| UBC26-Vector-F | CTCGAGTCTGGTAAAGAAACCGC | Replace UBC8 |
| UBC26-Vector-R | CGATATCCAATTGAGATCTGCCA | Replace UBC8 |
| UCIP2-Insert-F | GGATTTCAGAATTCGGATCCATGGCATATAGAAATACGGTATGCA | Replace ABI3 |
| UCIP2-Insert-R | ACGTCGTATGGGTAAGGCCTTCCACTTCTTCTTCCGGTTGAC | Replace ABI3 |
| UCIP2-Vector-F | AGGCCTTACCCATACGACGTT | Replace ABI3 |
| UCIP2-Vector-R | GGATCCGAATTCTGAAATCCTTC | Replace ABI3 |
| AvrPiz-t-Insert-F | GGATTTCAGAATTCGGATCCATGAGCTTCGTACAATGCAATCA | Replace ABI3 |
| AvrPiz-t-Insert-R | ACGTCGTATGGGTAAGGCCTTTGGCGCTGAGCCTGAGG | Replace ABI3 |
| E1-Cla1-F | CCATCGAT GGTACCCTCGAGTCTGGTAAAG | Delete AtUBA1 |
| E1-Cla1-R | CCATCGAT CGATATCCAATTGAGATCTG | Delete AtUBA1 |
| E2-Spe1-F | GGACTAGT CTCGAGTCTGGTAAAGAAAC | Delete UBC26 |
| E2-Spe1-R | GGACTAGT CGATATCCAATTGAGATCTG | Delete UBC26 |
| GST-AvrPiz-t-F | GGTTCCGCGTGGATCCCCGGGAATGAGCTTCGTACAATGCAATCA | Protein purification |
| GST-AvrPiz-t-R | TAAGCTTGAGCTCGAGTCGACCTATTGGCGCTGAGCCTGAG | Protein purification |
